# Supplementary material for: Combined Luteolin and Indole-3-Carbinol Synergistically Constrains ERα-Positive Breast Cancer by Dual Inhibiting Estrogen Receptor Alpha and Cyclin-Dependent Kinase 4/6 Pathway in Cultured Cells and Xenograft Mice
Source: Cancers (Basel). 2021 Apr 27;13(9):2116. doi: 10.3390/cancers13092116 (PMC8123907; doi:10.3390/cancers13092116)
Supplement: Supplementary file 1 [file cancers-13-02116-s001.zip › cancers-1165132-supplementary.pdf]

cancers

Article

Supplementary Material: Combined Luteolin and Indole-3-carbinol Synergistically Constrains ERα-positive Breast Cancer by Dual Inhibiting Estrogen Receptor alpha and Cyclin-dependent Kinase 4/6 in Cultured Cells and Xenograft Mice

Xiaoyong Wang, Lijuan Zhang, Qi Dai, Hongzong Si, Longyun Zhang, Sakina E Eltom and Hongwei Si

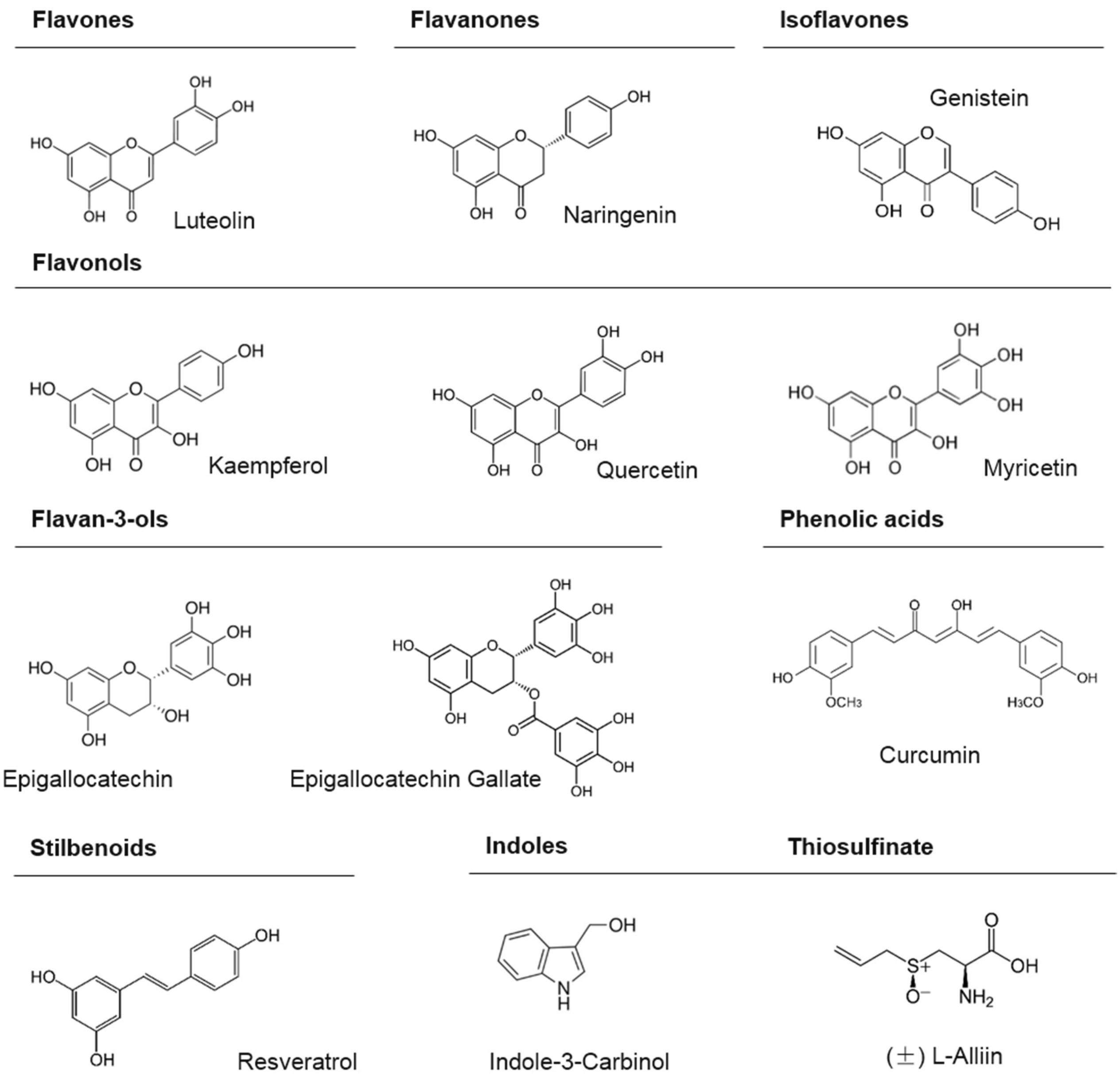

Figure S1. Structure of selected phytochemicals for initial dose-response screening.

Table S1. Dose-response metrics of selected phytochemicals in MCF7 and MDA-MB-231 cells were determined using non-linear regression curve fitting. EC, epigallocatechin; EGCG, epigallocatechin gallate; I3C, indole-3-carbinol; nd, not detected.

**Phyto-chemicals**	**MCF7**			**MDA-MB-231**		
	**EC_{20} (μM)**	**EC_{50} (μM)**	**E_{80} (μM)**	**EC_{20} (μM)**	**EC_{50} (μM)**	**E_{80} (μM)**
Luteolin	29.92±4.25	49.68±3.49	82.5±7.63	18.29±4.12	34.31±2.57	64.35±5.42
Myricetin	47.22±3.64	75.35±7.57	120.24±10.89	30.76±3.34	57.22±4.26	106.47±11.15
Quercetin	92.85±13.26	147.80±7.89	235.28±9.41	49.66±6.25	114.40±8.33	263.55±17.21
Kaempferol	98.68±9.96	167.56±6.48	284.52±11.74	61.96±4.52	108.12±7.71	188.68±15.14
Naringenin	115.72±13.16	187.25±14.25	315.22±17.29	90.23±18.29	207.12±10.37	368.15±22.08
EC	88.56±13.85	127.26±8.51	217.54±20.47	95.42±7.88	135.28±17.94	245.77±17.14
EGCG	80.27±12.43	103.49±7.23	129.75±8.97	98.41±13.62	127.11±11.27	159.23±18.35
Genistein	69.95±14.34	117.14±8.89	139.28±16.36	72.54±9.21	133.42±9.24	142.38±16.17
Curcumin	35.58±3.96	46.22±6.31	60.04±5.14	25.30±4.35	33.16±3.42	43.46±5.29
Resveratrol	73.71±18.04	129.31±9.47	177.26±21.03	67.19±8.16	98.92±17.25	130.47±11.28
I3C	146.33±20.52	202.9±9.83	281.33±12.13	284.44±21.74	397.21±15.36	nd
(±) L-Alliin	128.62±21.33	237.31±18.45	317.61±24.32	133.62±12.07	378.26±28.41	nd

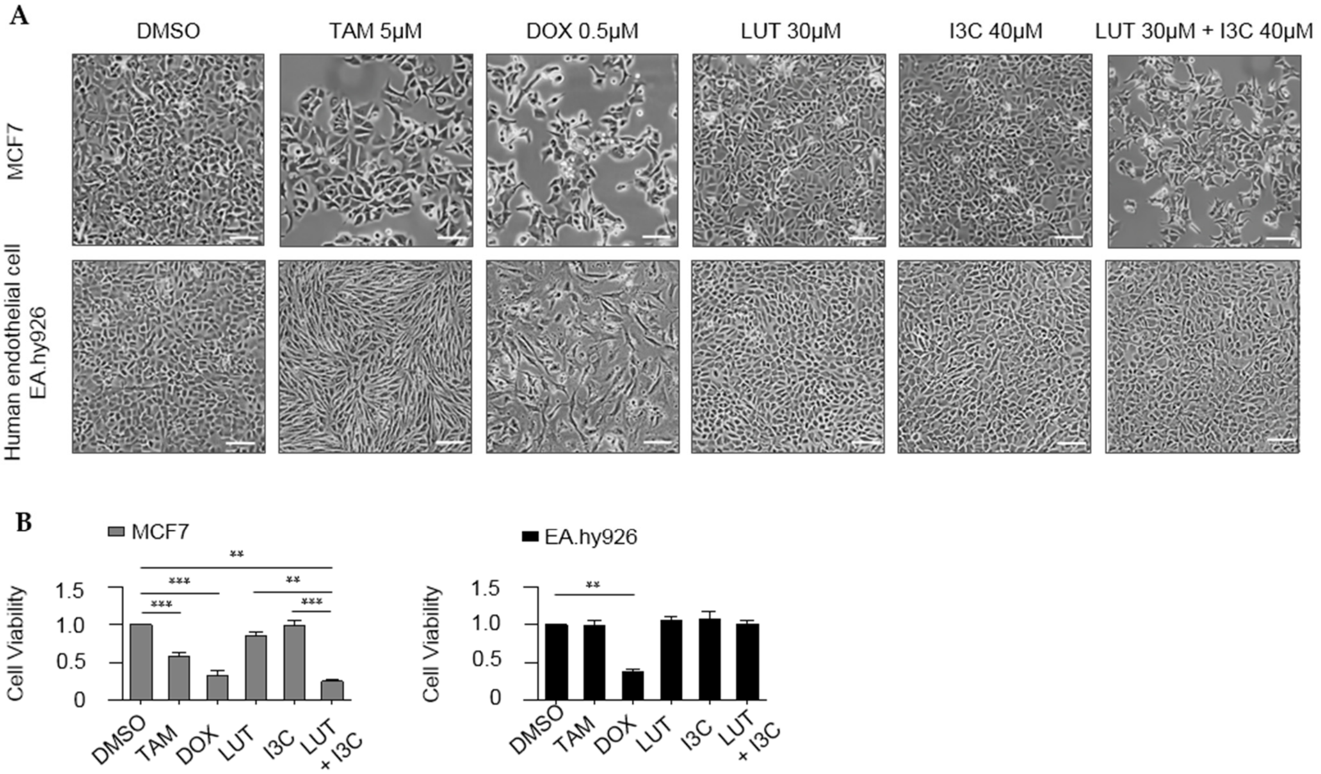

Figure S2. (A) Images of MCF7, T47D and human endothelial cells EA.hy926 treated with LUT (30 μM), I3C (40μM), L30I40 as well as Tamoxifen (TAM, 5μM) and Doxorubicin (DOX, 05μM) for 72h. The scale bar in the figure above represents 50μm. **(B)** Bar graph depicts cell viability of individual LUT, I3C, or combination L30I40, TAM and DOX in MCF7, T47D and EA.hy926 cells at 72h. Data are means ± SEM of at least three independent experiments performed in duplicate. * $P < 0.05$; ** $P < 0.01$; *** $P < 0.001$.

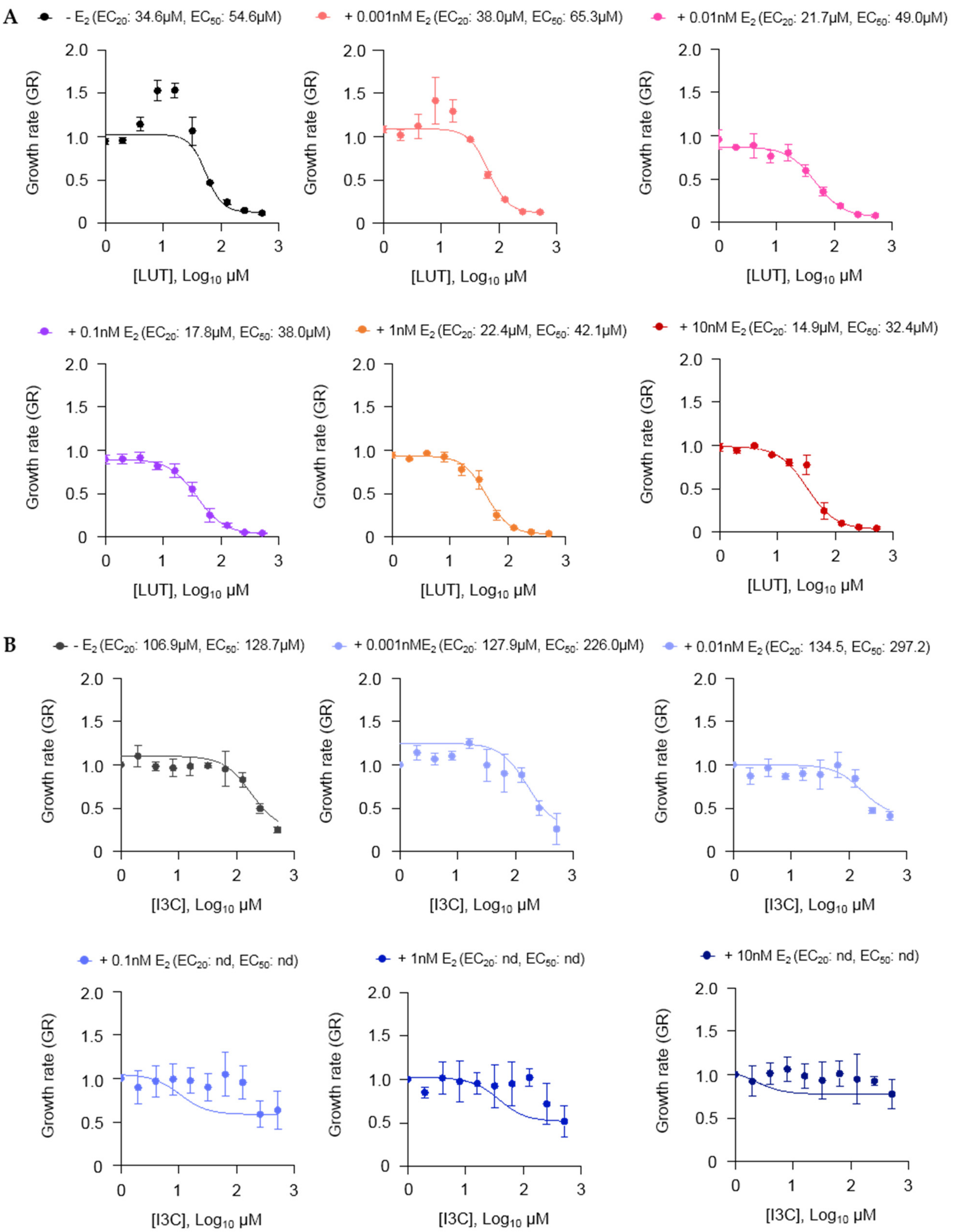

Figure S3. Inhibitory effect of Luteolin (LUT, A) and Indole-3-carbinol (I3C, B) on MCF7 cells growth rate (GR) in presence of different doses of estradiol E_2 (0-10nM).

Figure 2D

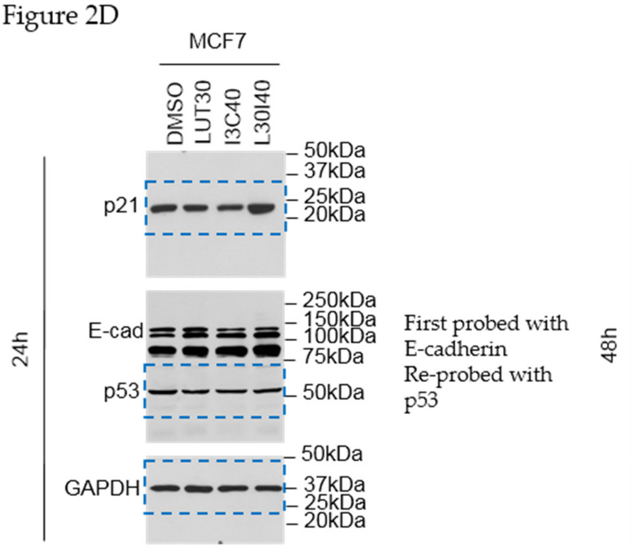

Intensity	DMSO	LUT30	I3C40	L30I40
p21	27848.581	23943.832	22280.510	36456.530
p53	40523.794	31972.530	33912.016	33785.338
GAPDH	35480.167	39438.924	38342.823	32981.439

Ratio	DMSO	LUT30	I3C40	L30I40
p21/GAPDH	1	0.773	0.740	1.408
p53/GAPDH	1	0.710	0.774	0.897

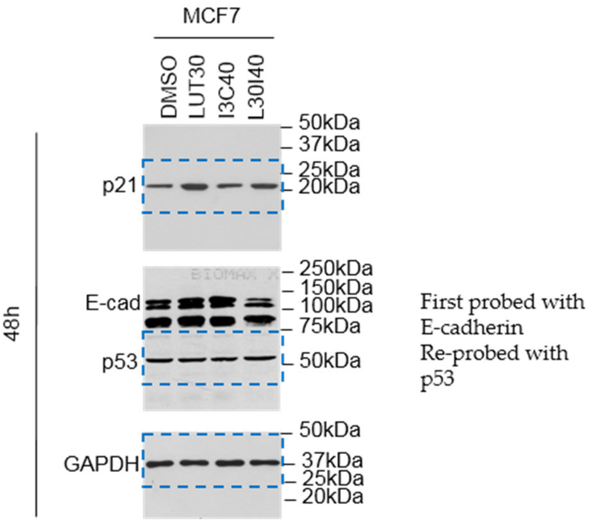

Intensity	DMSO	LUT30	I3C40	L30I40
p21	18062.317	27622.823	20973.681	24812.134
p53	37778.773	37999.551	35318.187	42481.329
GAPDH	35918.167	35009.995	40221.652	35752.874

Ratio	DMSO	LUT30	I3C40	L30I40
p21/GAPDH	1	1.569	1.037	1.380
p53/GAPDH	1	1.032	0.835	1.130

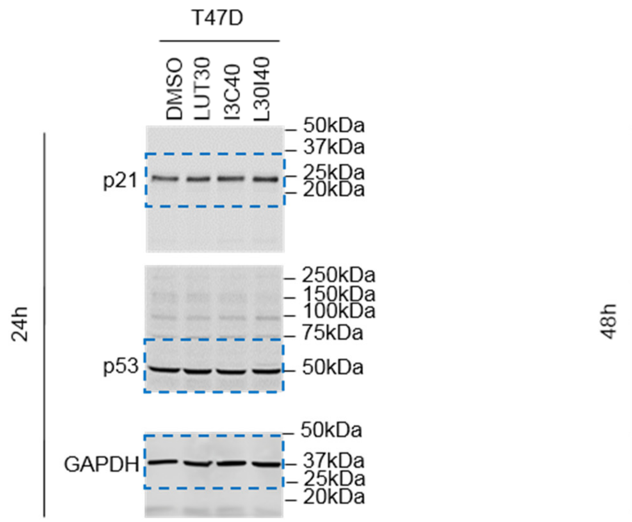

Intensity	DMSO	LUT30	I3C40	L30I40
p21	15176.175	15210.861	17618.004	18430.468
p53	22229.711	21791.225	20827.225	21123.447
GAPDH	22086.518	25998.317	26610.974	26193.439

Ratio	DMSO	LUT30	I3C40	L30I40
p21/GAPDH	1	0.851	0.964	1.024
p53/GAPDH	1	0.833	0.778	0.801

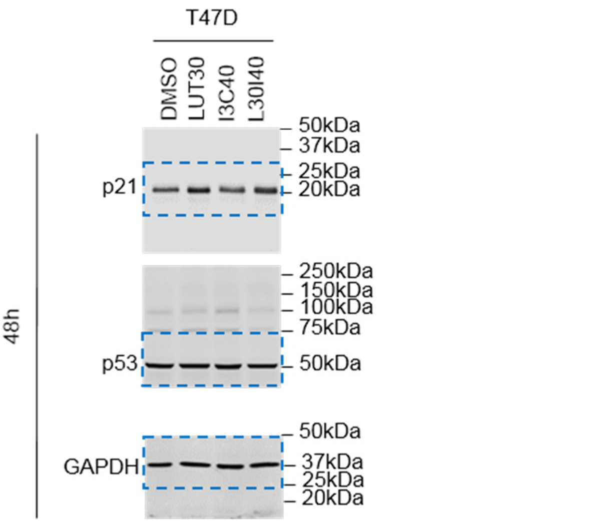

Intensity	DMSO	LUT30	I3C40	L30I40
p21	14181.397	17769.882	15099.296	17662.983
p53	22635.790	23090.912	22001.921	21082.134
GAPDH	21569.225	22302.468	23841.953	22997.711

Ratio	DMSO	LUT30	I3C40	L30I40
p21/GAPDH	1	1.212	0.963	1.168
p53/GAPDH	1	0.987	0.879	0.874

Figure 2E

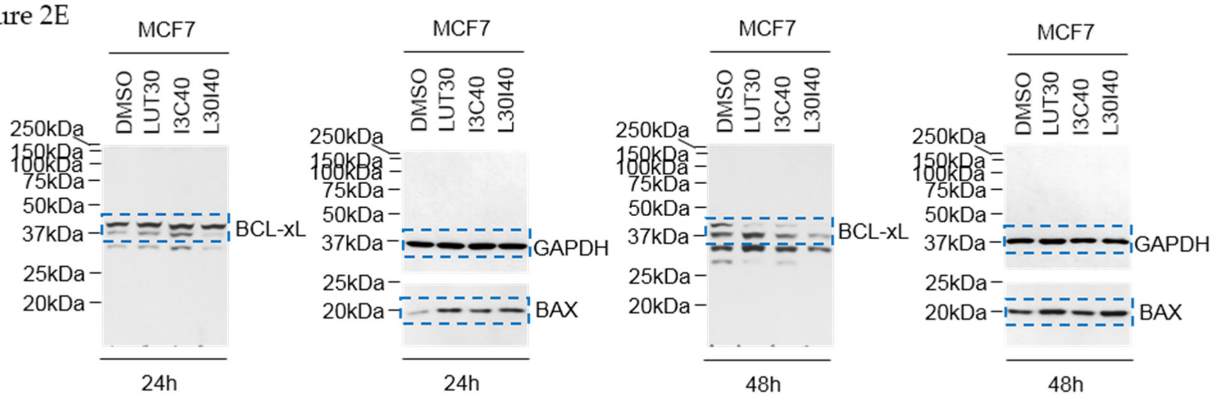

Intensity	DMSO	LUT30	I3C40	L30I40
BCL-xL	21501.146	30862.288	28241.894	18277.439
BAX	12052.217	15070.894	17882.874	22409.309
GAPDH	33868.045	38562.681	41763.167	39289.217

Ratio	DMSO	LUT30	I3C40	L30I40
BCL-xL/GAPDH	1	1.261	1.065	0.733
BAX/GAPDH	1	1.098	1.203	1.603

Intensity	DMSO	LUT30	I3C40	L30I40
BCL-xL	30144.288	27252.066	20934.853	7795.610
BAX	24939.288	42476.116	26918.388	34828.803
GAPDH	36787.681	40378.945	32632.530	36832.945

Ratio	DMSO	LUT30	I3C40	L30I40
BCL-xL/GAPDH	1	0.824	0.783	0.258
BAX/GAPDH	1	1.552	1.217	1.395

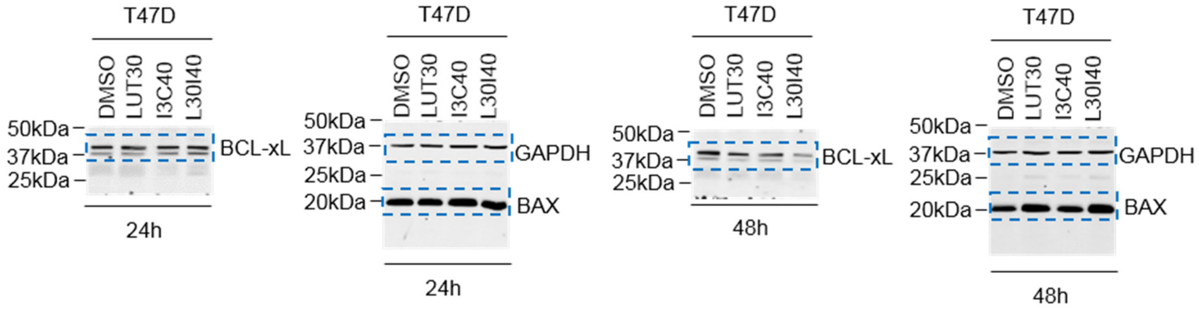

Intensity	DMSO	LUT30	I3C40	L30I40
BCL-xL	18633.740	20308.196	17948.933	19480.690
BAX	36394.782	33490.589	44548.317	38859.045
GAPDH	28973.803	30627.589	42689.640	36478.418

Ratio	DMSO	LUT30	I3C40	L30I40
BCL-xL/GAPDH	1	1.261	1.065	0.733
BAX/GAPDH	1	1.098	1.203	1.603

Intensity	DMSO	LUT30	I3C40	L30I40
BCL-xL	36571.439	24299.660	29340.924	15073.882
BAX	25756.690	39325.095	28539.903	40743.953
GAPDH	30853.874	32576.581	33044.104	33023.782

Ratio	DMSO	LUT30	I3C40	L30I40
BCL-xL/GAPDH	1	0.824	0.783	0.258
BAX/GAPDH	1	1.552	1.217	1.395

Figure 2F

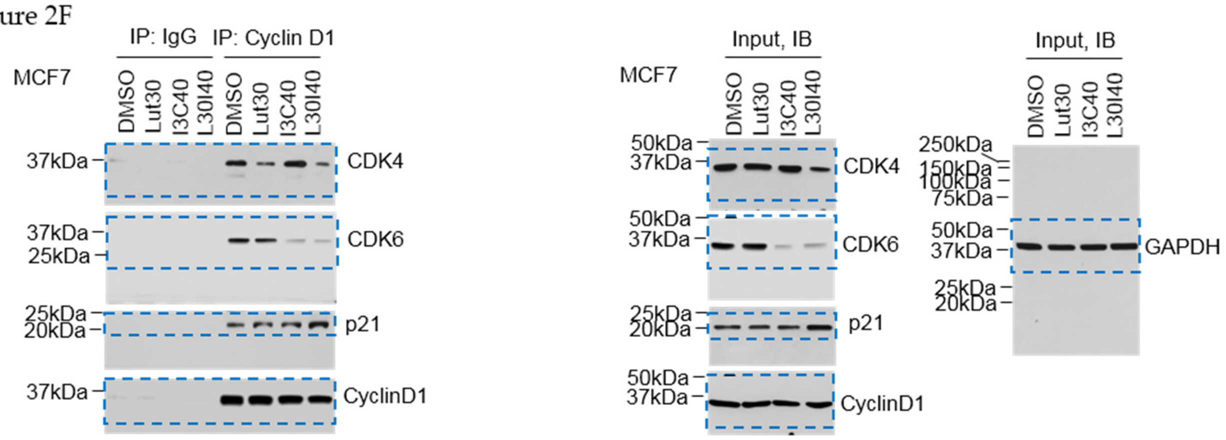

Intensity	DMSO	LUT30	I3C40	L30I40
CDK4	31002.652	13071.803	36938.874	9287.761
CDK6	21538.761	17695.418	4390.983	3115.305
p21	16319.489	18562.995	19973.803	32166.338
CyclinD1	38112.64	38934.761	35866.468	30371.054

Intensity	DMSO	LUT30	I3C40	L30I40
CDK4	36284.095	39038.024	37106.217	16743.267
CDK6	20610.296	21227.640	2741.305	4456.205
p21	25725.167	20460.004	23209.439	39680.782
CyclinD1	48240.803	42822.874	37474.388	37602.409
GAPDH	38099.167	35566.51	36876.246	40315.953

Ratio	DMSO	LUT30	I3C40	L30I40
CDK4	1	0.422	1.191	0.300
CDK6	1	0.822	0.204	0.145
p21	1	1.137	1.224	1.971
CyclinD1	1	1.022	0.941	0.797

Ratio	DMSO	LUT30	I3C40	L30I40
CDK4/GAPDH	1	1.153	1.057	0.436
CDK6/GAPDH	1	1.103	0.137	0.204
p21/GAPDH	1	0.852	0.932	1.458
CyclinD1/GAPDH	1	0.951	0.803	0.737

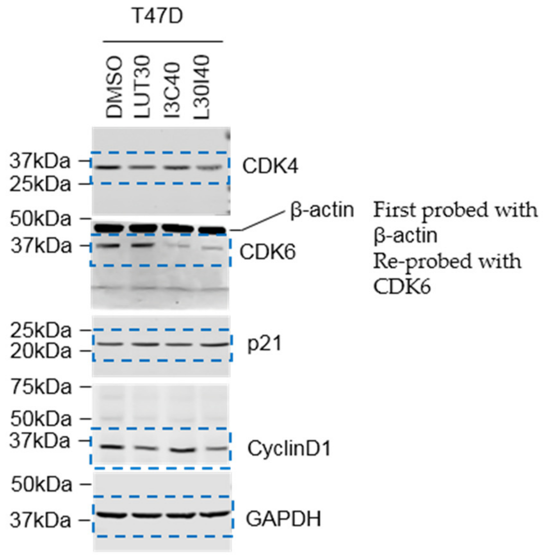

Intensity	DMSO	LUT30	I3C40	L30I40
CDK4	37296.489	27002.882	31570.409	24861.773
CDK6	23897.246	24478.610	10266.296	4428.841
p21	22301.711	27665.953	22498.66	30263.267
CyclinD1	35335.974	24120.832	35940.995	22002.125
GAPDH	56928.309	51296.288	52558.43	51866.894

Ratio	DMSO	LUT30	I3C40	L30I40
CDK4/GAPDH	1	0.803	0.917	0.732
CDK6/GAPDH	1	1.137	0.465	0.203
p21/GAPDH	1	1.377	1.093	1.489
CyclinD1/GAPDH	1	0.758	1.102	0.683

Figure S4. Uncropped western blot images related to Figure 2D, E and F.

Figure 3D

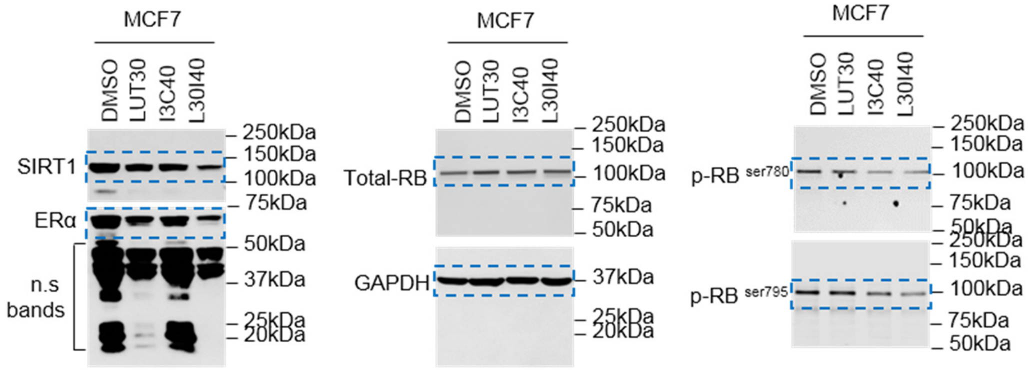

Intensity	DMSO	LUT30	I3C40	L30I40
SIRT1	43898.853	29936.489	30014.974	15265.539
ERα	42131.853	25620.681	29601.681	13809.175
p-RB S780	15604.125	10018.468	5950.811	5357.861
p-RB S795	27544.024	24522.51	15779.761	10483.368
Total-RB	35506.711	40952.296	38772.974	39265.64
GAPDH	38254.589	48669.095	41971.196	40941.146

Ratio	DMSO	LUT30	I3C40	L30I40
SIRT1/GAPDH	1.000	0.536	0.623	0.325
ERα/GAPDH	1.000	0.478	0.640	0.306
p-RB S780/GAPDH	1.000	0.505	0.348	0.321
p-RB S795/GAPDH	1.000	0.700	0.522	0.356
Total RB/GAPDH	1.000	0.907	0.995	1.033

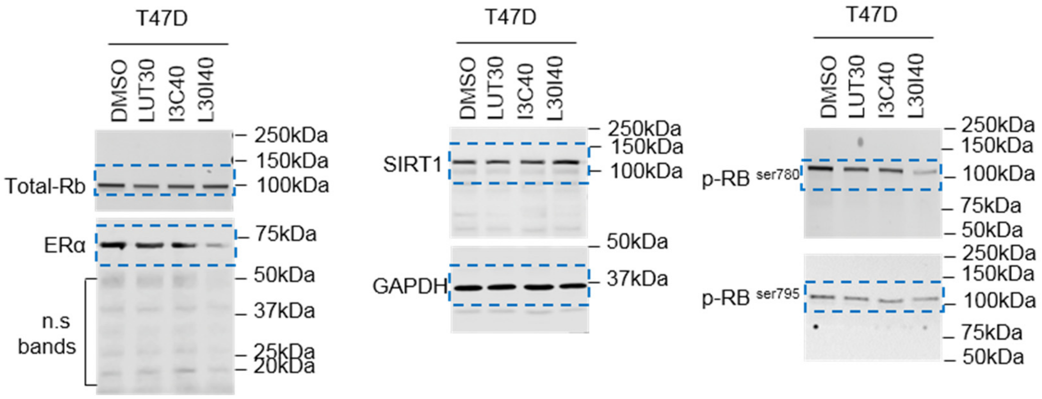

Intensity	DMSO	LUT30	I3C40	L30I40
SIRT1	27587.125	24814.589	27570.196	34985.853
ERα	29149.924	22598.359	20415.066	7780.468
p-RB S780	40705.69	26342.397	23567.761	8523.69
p-RB S795	20698.024	15019.903	16166.974	15682.489
Total-RB	41850.589	37147.953	35924.317	36685.368
GAPDH	38712.317	38221.317	38921.853	38153.125

Ratio	DMSO	LUT30	I3C40	L30I40
SIRT1	1.000	0.911	0.994	1.287
ERα	1.000	0.785	0.697	0.271
p-RB S780/GAPDH	1.000	0.655	0.576	0.212
p-RB S795/GAPDH	1.000	0.735	0.777	0.769
Total RB/GAPDH	1.000	0.899	0.854	0.889

Figure 3F (top panel)

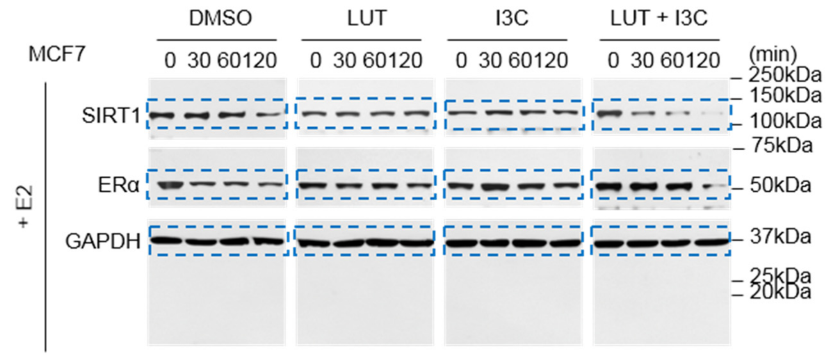

Intensity	DMSO			
Time (min)	0	30	60	120
SIRT1	27341.4	27836.8	27488.6	15558.8
ERα	34653.5	19576.2	19497.9	15790.9
GAPDH	39232.8	35062.4	39024.0	37418.4

Ratio	DMSO			
Time (min)	0	30	60	120
SIRT1/GAPDH	1	1.139	1.011	0.597
ERα/GAPDH	1	0.623	0.566	0.478

Intensity	I3C			
Time (min)	0	30	60	120
SIRT1	30636.8	37667.8	29755.4	26052.5
ERα	28190.2	38121.1	28849.6	22837.0
GAPDH	41613.2	39383.0	38581.9	37508.5

Ratio	I3C			
Time (min)	0	30	60	120
SIRT1/GAPDH	1	1.299	1.048	0.943
ERα/GAPDH	1	1.429	1.104	0.899

Intensity	LUT			
Time (min)	0	30	60	120
SIRT1	26438.2	27252.5	26190.7	31482.6
ERα	36771.5	28327.8	31013.6	20861.2
GAPDH	38301.9	36958.7	40953.7	39440.8

Ratio	LUT			
Time (min)	0	30	60	120
SIRT1/GAPDH	1	1.068	0.926	1.156
ERα/GAPDH	1	0.798	0.789	0.551

Intensity	LUT + I3C			
Time (min)	0	30	60	120
SIRT1	32466.3	14468.4	9080.6	2348.2
ERα	34393.9	37338.1	31202.1	7905.3
GAPDH	42003.9	42435.5	39618.1	42413.2

Ratio	LUT + I3C			
Time (min)	0	30	60	120
SIRT1/GAPDH	1	0.441	0.297	0.072
ERα/GAPDH	1	1.075	0.962	0.228

Figure 3F (bottom panel)

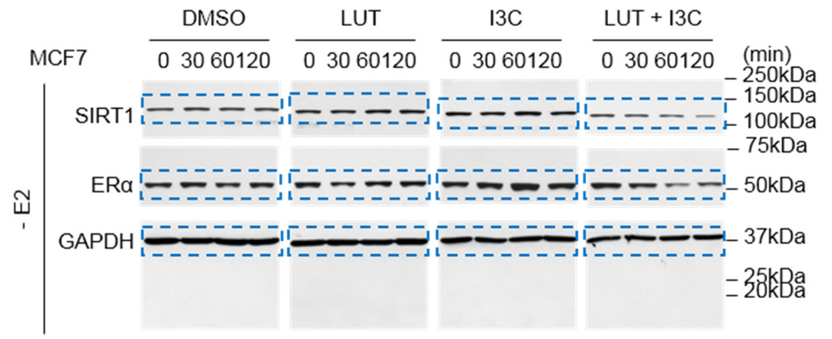

Intensity	DMSO			
Time (min)	0	30	60	120
SIRT1	33411.1	35367.8	30928.4	28058.0
ERα	34218.3	34723.8	25524.9	28521.7
GAPDH	48808.4	42936.1	47091.9	41028.2

Ratio	DMSO			
Time (min)	0	30	60	120
SIRT1/GAPDH	1	1.203	0.959	0.999
ERα/GAPDH	1	1.154	0.773	0.992

Intensity	I3C			
Time (min)	0	30	60	120
SIRT1	32784.7	27923.1	37807.1	29734.2
ERα	25171.5	28639.7	38856.1	29227.3
GAPDH	44013.2	39792.3	41424.7	42630.3

Ratio	I3C			
Time (min)	0	30	60	120
SIRT1/GAPDH	1	0.942	1.225	0.936
ERα/GAPDH	1	1.258	1.640	1.199

Intensity	LUT			
Time (min)	0	30	60	120
SIRT1	29584.4	28677.5	34975.6	34028.4
ERα	34613.9	22688.8	32430.3	34622.0
GAPDH	44779.9	43086.6	40350.3	46303.4

Ratio	LUT			
Time (min)	0	30	60	120
SIRT1/GAPDH	1	1.007	1.312	1.112
ERα/GAPDH	1	0.681	1.040	0.967

Intensity	LUT + I3C			
Time (min)	0	30	60	120
SIRT1	34407.7	28053.9	25218.5	15122.9
ERα	39893.7	28251.9	13306.9	17045.9
GAPDH	28106.8	27984.7	27390.4	27646.7

Ratio	LUT + I3C			
Time (min)	0	30	60	120
SIRT1/GAPDH	1	0.819	0.752	0.447
ERα/GAPDH	1	0.711	0.342	0.434

Figure 3G

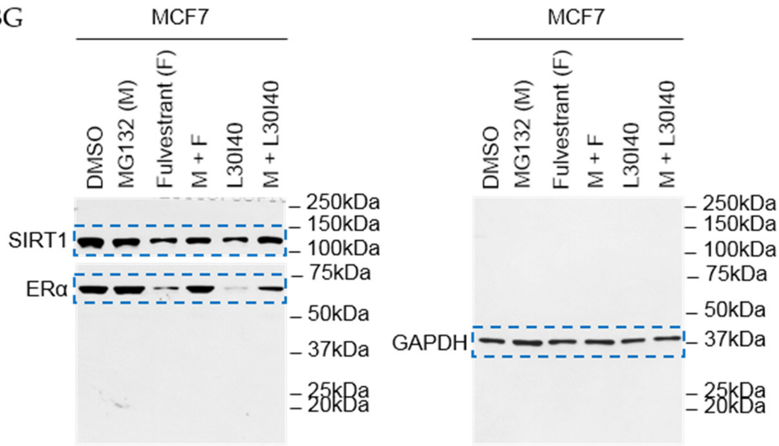

Intensity	DMSO	MG132	Ful	M + F	L30I40	M + L30I40
SIRT1	23073.9	22587.4	10363.8	15668.6	9419.6	14518.7
ERα	26209.9	28450.7	7769.3	21893.2	1955.3	11483.8
GAPDH	20484.2	22734.2	21583.5	24358.5	16698.1	16872.4

Intensity	DMSO	MG132	Ful	M + F	L30I40	M + L30I40
SIRT1/GAPDH	1	0.882	0.426	0.571	0.501	0.764
ERα/GAPDH	1	0.978	0.281	0.702	0.092	0.532

Figure 5. Uncropped western blot images related to Figure 3D, F and G.

Figure 4E MCF7 xenografts

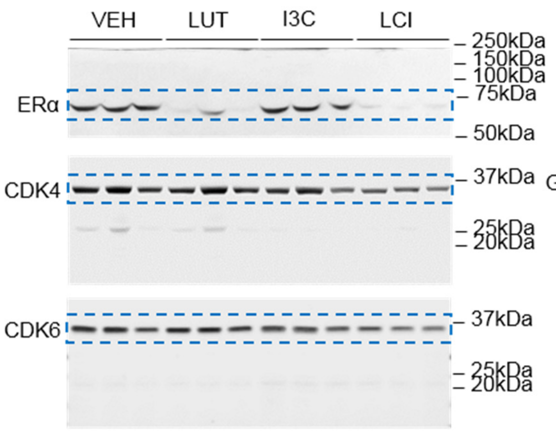

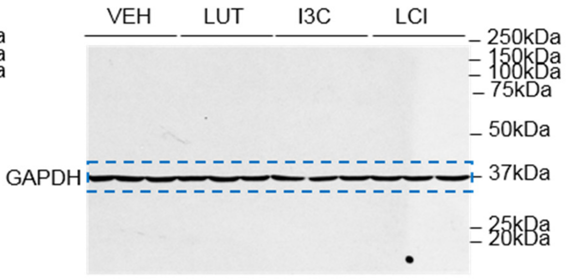

Intensity		VEH	
Mice	1	2	3
ERα	31580.9	31750.8	31957.2
CDK4	10983.8	12942.1	7834.8
CDK6	18602.5	18737.5	12503.7
GAPDH	33225.7	33365.6	34077.9

Ratio		VEH	
Mice	fold	se	sd
ERα/GAPDH	1	0.008	0.005
CDK4/GAPDH	1	0.253	0.146
CDK6/GAPDH	1	0.200	0.116

Intensity		LUT	
Mice	1	2	3
ERα	10971.4	20011.9	7246.7
CDK4	8980.1	13144.1	8597.0
CDK6	17307.1	17112.7	14391.3
GAPDH	32863.3	34445.6	28243.9

Ratio		LUT	
Mice	fold	se	sd
ERα/GAPDH	0.412	0.179	0.103
CDK4/GAPDH	1.011	0.176	0.102
CDK6/GAPDH	1.067	0.046	0.027

Intensity		I3C	
Mice	1	2	3
ERα	34066.6	31914.8	22363.1
CDK4	7968.2	10974.6	5577.5
CDK6	15942.3	15618.3	11987.0
GAPDH	31277.7	25634.4	27881.9

Ratio		I3C	
Mice	fold	se	sd
ERα/GAPDH	1.104	0.237	0.137
CDK4/GAPDH	0.931	0.377	0.217
CDK6/GAPDH	1.030	0.236	0.136

Intensity		LUT + I3C	
Mice	1	2	3
ERα	5056.3	1546.2	2241.3
CDK4	6014.3	5843.2	4477.9
CDK6	11469.0	8802.1	8917.2
GAPDH	29016.2	23918.8	24650.2

Ratio		LUT + I3C	
Mice	fold	se	sd
ERα/GAPDH	0.116	0.060	0.035
CDK4/GAPDH	0.668	0.100	0.058
CDK6/GAPDH	0.566	0.092	0.053

Figure S6. Uncropped western blot images related to Figure 4E.

Table S2. Pairewise Combination - LUT + I3C CI.

Cell Line	Cpd1	Cpd 1 Tested Con (µM)	Cpd2	Cpd 2 Tested Con (µM)	GR % mean	Fraction	CI
BT-549	Luteolin	0	I3C	0	100%	0%	N/A
BT-549	Luteolin	1	I3C	0	102%	-2%	N/A
BT-549	Luteolin	5	I3C	0	102%	-2%	N/A
BT-549	Luteolin	10	I3C	0	112%	-12%	N/A
BT-549	Luteolin	15	I3C	0	121%	-21%	N/A
BT-549	Luteolin	20	I3C	0	99%	1%	N/A
BT-549	Luteolin	25	I3C	0	86%	14%	N/A
BT-549	Luteolin	30	I3C	0	77%	23%	N/A
BT-549	Luteolin	0	I3C	10	113%	-13%	N/A
BT-549	Luteolin	1	I3C	10	98%	2%	N/A
BT-549	Luteolin	5	I3C	10	111%	-11%	N/A
BT-549	Luteolin	10	I3C	10	121%	-21%	N/A
BT-549	Luteolin	15	I3C	10	99%	1%	N/A
BT-549	Luteolin	20	I3C	10	102%	-2%	N/A
BT-549	Luteolin	25	I3C	10	92%	8%	1.188
BT-549	Luteolin	30	I3C	10	73%	27%	0.913
BT-549	Luteolin	0	I3C	20	113%	-13%	N/A
BT-549	Luteolin	1	I3C	20	116%	-16%	N/A
BT-549	Luteolin	5	I3C	20	101%	-1%	N/A
BT-549	Luteolin	10	I3C	20	106%	-6%	N/A
BT-549	Luteolin	15	I3C	20	120%	-20%	N/A
BT-549	Luteolin	20	I3C	20	98%	2%	N/A
BT-549	Luteolin	25	I3C	20	86%	14%	1.258
BT-549	Luteolin	30	I3C	20	80%	20%	1.141
BT-549	Luteolin	0	I3C	40	102%	-2%	N/A
BT-549	Luteolin	1	I3C	40	100%	0%	N/A
BT-549	Luteolin	5	I3C	40	102%	-2%	N/A
BT-549	Luteolin	10	I3C	40	118%	-18%	N/A
BT-549	Luteolin	15	I3C	40	102%	-2%	N/A
BT-549	Luteolin	20	I3C	40	99%	1%	N/A
BT-549	Luteolin	25	I3C	40	95%	5%	1.397
BT-549	Luteolin	30	I3C	40	83%	18%	1.250
BT-549	Luteolin	0	I3C	60	101%	-1%	N/A
BT-549	Luteolin	1	I3C	60	104%	-4%	N/A
BT-549	Luteolin	5	I3C	60	99%	1%	N/A
BT-549	Luteolin	10	I3C	60	113%	-13%	N/A
BT-549	Luteolin	15	I3C	60	93%	7%	1.089
BT-549	Luteolin	20	I3C	60	103%	-2%	N/A
BT-549	Luteolin	25	I3C	60	93%	7%	1.537
BT-549	Luteolin	30	I3C	60	74%	26%	1.143
BT-549	Luteolin	0	I3C	80	113%	-13%	N/A
BT-549	Luteolin	1	I3C	80	110%	-10%	N/A
BT-549	Luteolin	5	I3C	80	101%	-1%	N/A
BT-549	Luteolin	10	I3C	80	103%	-3%	N/A
BT-549	Luteolin	15	I3C	80	95%	5%	1.229
BT-549	Luteolin	20	I3C	80	92%	8%	1.452
BT-549	Luteolin	25	I3C	80	92%	8%	1.676
BT-549	Luteolin	30	I3C	80	81%	19%	1.467
BT-549	Luteolin	0	I3C	100	108%	-8%	N/A
BT-549	Luteolin	1	I3C	100	103%	-2%	N/A
BT-549	Luteolin	5	I3C	100	101%	-1%	N/A
BT-549	Luteolin	10	I3C	100	109%	-9%	N/A
BT-549	Luteolin	15	I3C	100	89%	12%	1.368
BT-549	Luteolin	20	I3C	100	98%	2%	N/A
BT-549	Luteolin	25	I3C	100	79%	21%	1.404
BT-549	Luteolin	30	I3C	100	77%	24%	1.576

BT-549	Luteolin	0	I3C	120	99%	1%	N/A
BT-549	Luteolin	1	I3C	120	103%	-3%	N/A
BT-549	Luteolin	5	I3C	120	93%	7%	1.060
BT-549	Luteolin	10	I3C	120	107%	-7%	N/A
BT-549	Luteolin	15	I3C	120	99%	2%	N/A
BT-549	Luteolin	20	I3C	120	85%	15%	1.341
BT-549	Luteolin	25	I3C	120	71%	29%	1.275
BT-549	Luteolin	30	I3C	120	73%	27%	1.419
MCF7	Luteolin	1	I3C	0	102%	-2%	N/A
MCF7	Luteolin	5	I3C	0	116%	-16%	N/A
MCF7	Luteolin	10	I3C	0	122%	-22%	N/A
MCF7	Luteolin	15	I3C	0	132%	-32%	N/A
MCF7	Luteolin	20	I3C	0	124%	-24%	N/A
MCF7	Luteolin	25	I3C	0	117%	-17%	N/A
MCF7	Luteolin	30	I3C	0	83%	17%	N/A
MCF7	Luteolin	0	I3C	10	99%	1%	N/A
MCF7	Luteolin	1	I3C	10	102%	-2%	N/A
MCF7	Luteolin	5	I3C	10	112%	-12%	N/A
MCF7	Luteolin	10	I3C	10	120%	-20%	N/A
MCF7	Luteolin	15	I3C	10	116%	-16%	N/A
MCF7	Luteolin	20	I3C	10	126%	-26%	N/A
MCF7	Luteolin	25	I3C	10	129%	-29%	N/A
MCF7	Luteolin	30	I3C	10	90%	10%	1.432
MCF7	Luteolin	0	I3C	20	101%	-1%	N/A
MCF7	Luteolin	1	I3C	20	112%	-12%	N/A
MCF7	Luteolin	5	I3C	20	102%	-2%	N/A
MCF7	Luteolin	10	I3C	20	108%	-8%	N/A
MCF7	Luteolin	15	I3C	20	93%	7%	0.840
MCF7	Luteolin	20	I3C	20	93%	7%	1.065
MCF7	Luteolin	25	I3C	20	87%	13%	1.290
MCF7	Luteolin	30	I3C	20	66%	34%	0.943
MCF7	Luteolin	0	I3C	40	102%	-2%	N/A
MCF7	Luteolin	1	I3C	40	103%	-2%	N/A
MCF7	Luteolin	5	I3C	40	85%	15%	0.440
MCF7	Luteolin	10	I3C	40	76%	24%	0.608
MCF7	Luteolin	15	I3C	40	63%	37%	0.567
MCF7	Luteolin	20	I3C	40	63%	37%	0.684
MCF7	Luteolin	25	I3C	40	66%	34%	0.926
MCF7	Luteolin	30	I3C	40	26%	74%	0.579
MCF7	Luteolin	0	I3C	60	104%	-4%	N/A
MCF7	Luteolin	1	I3C	60	93%	7%	0.541
MCF7	Luteolin	5	I3C	60	75%	25%	0.498
MCF7	Luteolin	10	I3C	60	63%	37%	0.559
MCF7	Luteolin	15	I3C	60	68%	32%	0.772
MCF7	Luteolin	20	I3C	60	53%	47%	0.698
MCF7	Luteolin	25	I3C	60	47%	53%	0.799
MCF7	Luteolin	30	I3C	60	25%	75%	0.577
MCF7	Luteolin	0	I3C	80	96%	4%	N/A
MCF7	Luteolin	1	I3C	80	101%	-1%	N/A
MCF7	Luteolin	5	I3C	80	65%	35%	0.551
MCF7	Luteolin	10	I3C	80	58%	42%	0.667
MCF7	Luteolin	15	I3C	80	55%	45%	0.696
MCF7	Luteolin	20	I3C	80	38%	62%	0.706
MCF7	Luteolin	25	I3C	80	25%	75%	0.587
MCF7	Luteolin	30	I3C	80	23%	78%	0.648
MCF7	Luteolin	0	I3C	100	90%	10%	N/A
MCF7	Luteolin	1	I3C	100	92%	8%	0.872
MCF7	Luteolin	5	I3C	100	62%	39%	0.659
MCF7	Luteolin	10	I3C	100	69%	31%	0.876

MCF7	Luteolin	15	I3C	100	51%	49%	0.795
MCF7	Luteolin	20	I3C	100	37%	63%	0.796
MCF7	Luteolin	25	I3C	100	28%	72%	0.773
MCF7	Luteolin	30	I3C	100	13%	87%	0.562
MCF7	Luteolin	0	I3C	120	73%	27%	N/A
MCF7	Luteolin	1	I3C	120	76%	25%	0.749
MCF7	Luteolin	5	I3C	120	69%	31%	0.859
MCF7	Luteolin	10	I3C	120	63%	37%	0.884
MCF7	Luteolin	15	I3C	120	49%	51%	0.893
MCF7	Luteolin	20	I3C	120	33%	67%	0.780
MCF7	Luteolin	25	I3C	120	29%	72%	0.854
MCF7	Luteolin	30	I3C	120	13%	87%	0.621
MD-MB-231	Luteolin	0	I3C	0	100%	0%	N/A
MD-MB-231	Luteolin	1	I3C	0	101%	-1%	N/A
MD-MB-231	Luteolin	5	I3C	0	112%	-12%	N/A
MD-MB-231	Luteolin	10	I3C	0	103%	-3%	N/A
MD-MB-231	Luteolin	15	I3C	0	93%	7%	N/A
MD-MB-231	Luteolin	20	I3C	0	87%	13%	N/A
MD-MB-231	Luteolin	25	I3C	0	83%	17%	N/A
MD-MB-231	Luteolin	30	I3C	0	73%	27%	N/A
MD-MB-231	Luteolin	0	I3C	10	115%	-15%	N/A
MD-MB-231	Luteolin	1	I3C	10	98%	2%	N/A
MD-MB-231	Luteolin	5	I3C	10	115%	-15%	N/A
MD-MB-231	Luteolin	10	I3C	10	98%	2%	N/A
MD-MB-231	Luteolin	15	I3C	10	112%	-12%	N/A
MD-MB-231	Luteolin	20	I3C	10	86%	14%	1.623
MD-MB-231	Luteolin	25	I3C	10	83%	17%	1.402
MD-MB-231	Luteolin	30	I3C	10	72%	28%	1.314
MD-MB-231	Luteolin	0	I3C	20	102%	-2%	N/A
MD-MB-231	Luteolin	1	I3C	20	112%	-12%	N/A
MD-MB-231	Luteolin	5	I3C	20	102%	-2%	N/A
MD-MB-231	Luteolin	10	I3C	20	104%	-4%	N/A
MD-MB-231	Luteolin	15	I3C	20	98%	2%	N/A
MD-MB-231	Luteolin	20	I3C	20	91%	9%	1.665
MD-MB-231	Luteolin	25	I3C	20	82%	18%	1.437
MD-MB-231	Luteolin	30	I3C	20	72%	28%	1.345
MD-MB-231	Luteolin	0	I3C	40	123%	-23%	N/A
MD-MB-231	Luteolin	1	I3C	40	104%	-3%	N/A
MD-MB-231	Luteolin	5	I3C	40	101%	-1%	N/A
MD-MB-231	Luteolin	10	I3C	40	108%	-8%	N/A
MD-MB-231	Luteolin	15	I3C	40	104%	-3%	N/A
MD-MB-231	Luteolin	20	I3C	40	102%	-2%	N/A
MD-MB-231	Luteolin	25	I3C	40	79%	21%	1.508
MD-MB-231	Luteolin	30	I3C	40	70%	31%	1.407
MD-MB-231	Luteolin	0	I3C	60	104%	-3%	N/A
MD-MB-231	Luteolin	1	I3C	60	116%	-16%	N/A
MD-MB-231	Luteolin	5	I3C	60	102%	-1%	N/A
MD-MB-231	Luteolin	10	I3C	60	102%	-2%	N/A
MD-MB-231	Luteolin	15	I3C	60	98%	2%	N/A
MD-MB-231	Luteolin	20	I3C	60	97%	3%	N/A
MD-MB-231	Luteolin	25	I3C	60	82%	18%	1.578
MD-MB-231	Luteolin	30	I3C	60	67%	33%	1.469
MD-MB-231	Luteolin	0	I3C	80	102%	-2%	N/A
MD-MB-231	Luteolin	1	I3C	80	104%	-4%	N/A
MD-MB-231	Luteolin	5	I3C	80	112%	-12%	N/A
MD-MB-231	Luteolin	10	I3C	80	102%	-2%	N/A
MD-MB-231	Luteolin	15	I3C	80	93%	8%	1.527
MD-MB-231	Luteolin	20	I3C	80	86%	14%	1.922
MD-MB-231	Luteolin	25	I3C	80	74%	26%	1.317

MD-MB-231	Luteolin	30	I3C	80	69%	32%	1.531
MD-MB-231	Luteolin	0	I3C	100	101%	0%	N/A
MD-MB-231	Luteolin	1	I3C	100	101%	-1%	N/A
MD-MB-231	Luteolin	5	I3C	100	112%	-12%	N/A
MD-MB-231	Luteolin	10	I3C	100	95%	6%	1.217
MD-MB-231	Luteolin	15	I3C	100	102%	-2%	N/A
MD-MB-231	Luteolin	20	I3C	100	93%	7%	2.007
MD-MB-231	Luteolin	25	I3C	100	75%	25%	1.378
MD-MB-231	Luteolin	30	I3C	100	61%	39%	1.329
MD-MB-231	Luteolin	0	I3C	120	111%	-11%	N/A
MD-MB-231	Luteolin	1	I3C	120	113%	-13%	N/A
MD-MB-231	Luteolin	5	I3C	120	98%	2%	N/A
MD-MB-231	Luteolin	10	I3C	120	112%	-12%	N/A
MD-MB-231	Luteolin	15	I3C	120	99%	2%	N/A
MD-MB-231	Luteolin	20	I3C	120	82%	18%	1.515
MD-MB-231	Luteolin	25	I3C	120	70%	30%	1.440
MD-MB-231	Luteolin	30	I3C	120	64%	36%	1.384
T47D	Luteolin	30	I3C	120	0.198	80%	0.683
T47D	Luteolin	30	I3C	100	0.218	78%	0.637
T47D	Luteolin	25	I3C	120	0.287	71%	0.716
T47D	Luteolin	30	I3C	80	0.304	70%	0.678
T47D	Luteolin	25	I3C	100	0.319	68%	0.659
T47D	Luteolin	30	I3C	60	0.322	68%	0.692
T47D	Luteolin	25	I3C	80	0.346	65%	0.603
T47D	Luteolin	30	I3C	40	0.352	65%	0.564
T47D	Luteolin	20	I3C	120	0.386	61%	0.731
T47D	Luteolin	25	I3C	60	0.417	58%	0.610
T47D	Luteolin	20	I3C	80	0.417	58%	0.596
T47D	Luteolin	20	I3C	100	0.429	57%	0.664
T47D	Luteolin	20	I3C	60	0.502	50%	0.588
T47D	Luteolin	25	I3C	40	0.521	48%	0.597
T47D	Luteolin	15	I3C	120	0.598	40%	0.839
T47D	Luteolin	20	I3C	40	0.617	38%	0.564
T47D	Luteolin	15	I3C	100	0.627	37%	0.746
T47D	Luteolin	10	I3C	120	0.659	34%	0.864
T47D	Luteolin	30	I3C	20	0.693	31%	0.726
T47D	Luteolin	5	I3C	120	0.722	28%	0.762
T47D	Luteolin	10	I3C	100	0.734	27%	0.755
T47D	Luteolin	15	I3C	60	0.751	25%	0.638
T47D	Luteolin	15	I3C	80	0.762	24%	0.883
T47D	Luteolin	15	I3C	40	0.813	19%	0.612
T47D	Luteolin	10	I3C	80	0.824	18%	0.770
T47D	Luteolin	20	I3C	20	0.825	18%	0.590
T47D	Luteolin	25	I3C	20	0.841	16%	0.703
T47D	Luteolin	10	I3C	40	0.893	11%	0.636
T47D	Luteolin	5	I3C	100	0.896	10%	1.063
T47D	Luteolin	10	I3C	60	0.915	9%	0.822
T47D	Luteolin	5	I3C	40	0.915	9%	0.504
T47D	Luteolin	5	I3C	60	0.925	8%	0.690
T47D	Luteolin	1	I3C	20	0.939	6%	0.213
T47D	Luteolin	20	I3C	10	0.953	5%	0.621
T47D	Luteolin	5	I3C	10	0.958	4%	N/A
T47D	Luteolin	30	I3C	0	0.971	3%	N/A
T47D	Luteolin	1	I3C	120	0.974	3%	N/A
T47D	Luteolin	5	I3C	80	0.978	2%	N/A
T47D	Luteolin	0	I3C	20	0.980	2%	N/A
T47D	Luteolin	0	I3C	100	0.983	2%	N/A
T47D	Luteolin	25	I3C	10	0.987	1%	N/A
T47D	Luteolin	0	I3C	80	0.993	1%	N/A

T47D	Luteolin	0	I3C	40	0.997	0%	N/A
T47D	Luteolin	1	I3C	100	0.997	0%	N/A
T47D	Luteolin	0	I3C	120	1.012	-1%	N/A
T47D	Luteolin	25	I3C	0	1.017	-2%	N/A
T47D	Luteolin	15	I3C	20	1.017	-2%	N/A
T47D	Luteolin	10	I3C	20	1.021	-2%	N/A
T47D	Luteolin	1	I3C	40	1.025	-2%	N/A
T47D	Luteolin	10	I3C	10	1.032	-3%	N/A
T47D	Luteolin	0	I3C	10	1.051	-5%	N/A
T47D	Luteolin	0	I3C	60	1.058	-6%	N/A
T47D	Luteolin	1	I3C	0	1.068	-7%	N/A
T47D	Luteolin	20	I3C	0	1.073	-7%	N/A
T47D	Luteolin	15	I3C	10	1.104	-10%	N/A
T47D	Luteolin	30	I3C	10	1.134	-13%	N/A
T47D	Luteolin	1	I3C	60	1.143	-14%	N/A
T47D	Luteolin	1	I3C	10	1.149	-15%	N/A
T47D	Luteolin	15	I3C	0	1.151	-15%	N/A
T47D	Luteolin	1	I3C	80	1.155	-16%	N/A
T47D	Luteolin	5	I3C	0	1.160	-16%	N/A
T47D	Luteolin	5	I3C	20	1.204	-20%	N/A
T47D	Luteolin	10	I3C	0	1.354	-35%	N/A

CellLine: cell line clean name. Cpd1: compound 1. Cpd2: compound 2. GR % mean: average of GR % from three independent experiments. CI: Combination index. Multiple dose (lower than EC20): combined Cpd1 and Cpd2 using EC20 for each Cpd at ratio 1:1. Fraction: Fraction of cells were inhibited by the combination, we calculate the CI value when fraction > 4%.
